# Performance status and acute organ dysfunction influence hospital mortality in critically ill patients with cancer and suspected infection: a retrospective cohort analysis

**DOI:** 10.5935/0103-507X.20210038

**Published:** 2021

**Authors:** Ramon Teixeira Costa, Fernando Godinho Zampieri, Pedro Caruso, Antonio Paulo Nassar Júnior

**Affiliations:** 1 Intensive Care Unit, A.C. Camargo Cancer Center - São Paulo (SP), Brazil.; 2 Research Institute, HCor-Hospital do Coração - São Paulo (SP), Brazil.

**Keywords:** Neoplasms, Critical care, Critical illness, Sepsis, Organ dysfunction score, Hospital mortality, Neoplasias, Cuidados críticos, Estado terminal, Sepse, Escores de disfunção orgânica, Mortalidade hospitalar

## Abstract

**Objective:**

To evaluate how performance status impairment and acute organ dysfunction influence hospital mortality in critically ill patients with cancer who were admitted with suspected sepsis.

**Methods:**

Data were obtained from a retrospective cohort of patients, admitted to an intensive care unit, with cancer and with a suspected infection who received parenteral antibiotics and underwent the collection of bodily fluid samples. We used logistic regression with hospital mortality as the outcome and the Sequential Organ Failure Assessment score, Eastern Cooperative Oncology Group status, and their interactions as predictors.

**Results:**

Of 450 patients included, 265 (58.9%) died in the hospital. For patients admitted to the intensive care unit with lower Sequential Organ Failure Assessment (≤ 6), performance status impairment influenced the in-hospital mortality, which was 32% among those with no and minor performance status impairment and 52% among those with moderate and severe performance status impairment, p < 0.01. However, for those with higher Sequential Organ Failure Assessment (> 6), performance status impairment did not influence the in-hospital mortality (73% among those with no and minor impairment and 84% among those with moderate and severe impairment; p = 0.1).

**Conclusion:**

Performance status impairment seems to influence hospital mortality in critically ill cancer patients with suspected sepsis when they have less severe acute organ dysfunction at the time of intensive care unit admission.

## Abstract (pt)

**Objetivo:**

Avaliar como a funcionalidade e a disfunção orgânica aguda influenciam a mortalidade hospitalar de pacientes oncológicos admitidos com suspeita de sepse.

**Métodos:**

Os dados foram obtidos de uma coorte retrospectiva de pacientes oncológicos com suspeita de infecção admitidos em uma unidade de terapia intensiva. Estes receberam antibióticos por via parenteral e tiveram suas culturas coletadas. Utilizamos uma regressão logística, para avaliar a mortalidade hospitalar como desfecho, *Sequential Organ Failure Assessment e Eastern Cooperative Oncology Group* como preditores, além de suas interações.

**Resultados:**

Dentre os 450 pacientes incluídos, 265 (58,9%) morreram no hospital. Para os pacientes admitidos na unidade de terapia intensiva com *Sequential Organ Failure Assessment* baixo (≤ 6), o comprometimento da funcionalidade influenciou a mortalidade hospitalar, que foi de 32% entre os pacientes sem comprometimento ou com comprometimento mínimo da funcionalidade e 52% entre os pacientes com comprometimento moderado e grave (p < 0,01). Nos pacientes com *Sequential Organ Failure Assessment* elevado (> 6), a funcionalidade não influenciou a mortalidade hospitalar (73% entre os pacientes sem comprometimento ou com comprometimento mínimo, e 84% entre os pacientes com comprometimento moderado e grave; p = 0,1).

**Conclusão:**

O comprometimento da funcionalidade parece influenciar a mortalidade hospitalar de pacientes oncológicos com suspeita de sepse sem disfunções orgânicas agudas ou que apresentem disfunções leves no momento da admissão na unidade de terapia intensiva.

## INTRODUCTION

More than 20% of patients admitted to intensive care units (ICUs) have cancer, and sepsis is a leading reason for these admissions.^(1)^ Despite aggressive measures, sepsis continues to be a major cause of death among critically ill patients with cancer, with a hospital mortality rate as high as 60%.^(1,2)^ Indeed, some of these patients would not benefit from aggressive supportive care, which would cause them to spend their last days of life away from their relatives and cause them to have invasive and painful procedures.^(3)^

Outcomes for critically ill patients with cancer depend on the patient’s baseline characteristics, cancer status, and severity of organ dysfunction.^(4)^ Among measures of baseline health status, performance status (PS) is a relevant prognostic measure for critically ill patients, independent of age and comorbidities.^(1,4)^ The associations between PS impairment and ICU patient morbidity and mortality have been extensively demonstrated.^(1,4,5)^ However, the interaction between the PS and acute organ dysfunction has not been documented in cancer patients admitted for sepsis.

The aim of this study was to evaluate how PS impairment and acute organ dysfunction influence hospital mortality in critically ill patients with cancer who were admitted with suspected sepsis. Our hypothesis was that the influence of the PS on hospital mortality would differ according to the severity of acute organ dysfunction. The assessment of PS could be appropriate in the therapeutic decision-making of septic patients admitted to the ICU.

## METHODS

This study involved a secondary analysis of data from a retrospective cohort study of patients, with suspected infection, admitted to a 55-bed ICU in a cancer center, between January 2014 and January 2015. The study protocol was approved by the Ethics Committee of the A.C. Camargo Cancer Center. Due to the retrospective and observational nature of the present study, patient identification was not involved, and the requirement for Informed Consent was waived.

Data were retrieved from a prospectively collected database of ICU admissions. The following clinical data were collected: patient age and sex, PS (defined according to Eastern Cooperative Oncology Group - ECOG criteria)^(6)^ one week prior to hospital admission, worst and best vital signs during the first day in the ICU, lactate level on ICU admission, Simplified Acute Physiological Score 3 (SAPS 3), first-day total Sequential Organ Failure Assessment (SOFA) score, source of infection, etiological diagnosis, comorbidities, ICU length of stay, organ support measures required during the ICU stay (e.g., vasopressors, mechanical ventilation, and renal replacement therapy), and ICU and hospital mortality. The ECOG criteria define categories of PS: zero if fully active; one if indicating some restriction in the performance of physically strenuous activity, but the patient is still ambulatory and able to carry out work of a light or sedentary nature; two if the patient is ambulatory and up and about for > 50% of waking hours, capable of all self-care, but unable to carry out any work activities; three if capable of limited self-care and confined to a bed or chair for more than 50% of waking hours; and four if completely disabled, unable to carry out any self-care and totally confined to a bed or chair.^(6)^ The PS classification was based on the family member report at the time of ICU admission. No PS data were missing from the database.

The primary outcome was in-hospital mortality. Patients were defined as eligible for withdrawal or withholding regardless of their discharge from the ICU and were considered in the end-of-life decisions.

We included all patients with active cancer admitted for suspected infection who had received prescriptions for parenteral antibiotics and had undergone the collection of bodily fluid samples (e.g., blood, urine, cerebrospinal fluid, tracheal aspirate, or bronchoalveolar lavage) for bacterial culture analysis at the time of or immediately prior to admission. We excluded patients with suspected ICU-acquired infections.^(7)^ The patients were divided into groups according to their PS impairment.

### Statistical analysis

Data are presented as numbers and percentages, means and standard deviations, medians and interquartile ranges, or proportions with 95% confidence intervals. Normally distributed continuous variables were compared using the Student’s t test and analysis of variance, and nonnormally distributed continuous variables were compared using the Mann-Whitney rank sum or Kruskal-Wallis test.

Two regression models were applied. First, we performed a logistic regression analysis using hospital mortality as the outcome between the ECOG scale and SOFA score and between the ECOG scale and SAPS 3 score to evaluate their interaction as predictors. Second, we constructed a decision algorithm using the nonparametric decision tree method. The study population was classified into branch-like segments that construct an inverted tree with a root node, internal nodes, and leaf nodes. The branch choices were based on the risk indicator with the minimum p-value from the chi-square statistic of that division. The branching was limited for the three levels. For this analysis, we considered the ECOG and SOFA as continuous variables and hospital mortality as a single binary target.

We used the Spearman rank correlation coefficient to define the effects of the SOFA score on PS impairment.

**Figure 1:**
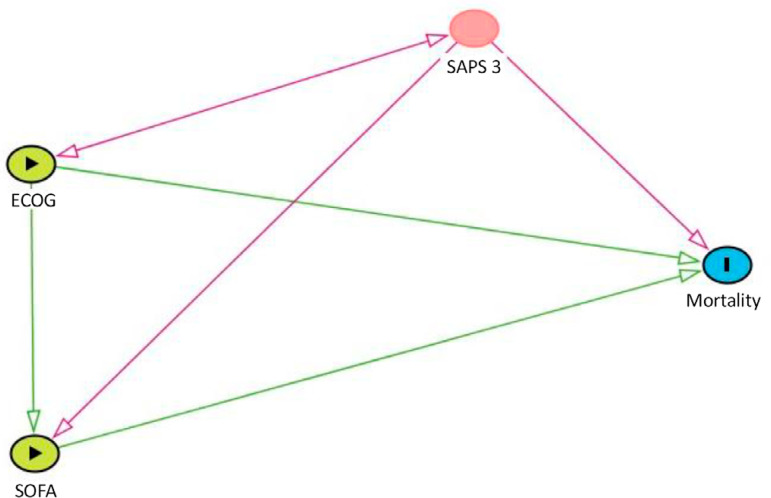
The influence of the performance status and Sequential Organ Failure Assessment score in cancer patients admitted to the intensive care unit with sepsis. ECOG - Eastern Cooperative Oncology Group; SAPS 3 - Simplified Acute Physiologic Score 3; SOFA - Sequential Organ Failure Assessment.

We made a directed acyclic graph (DAG) representing the influences of PS and the SOFA score in cancer patients admitted to the ICU with sepsis (Figure 1).

The results are presented as the probability of death according to the predictors. We considered a p value of < 0.05 to be significant for all analyses.

## RESULTS

In total, 485 patients were admitted to our ICU with suspected infection. We excluded 35 (7.2%) patients because they did not have active cancer. Thus, 450 patients were included in the study.

The main patient characteristics are presented in table 1. Most patients had metastatic solid tumors, and the main sources of infection were pulmonary and abdominal. The ICU and hospital mortality rates were 38.9% and 58.9%, respectively. End-of-life decisions were made in the ICU for 99 (22%) patients. According to the Sepsis-3 definitions,^(7)^ most (n = 288, 64%) patients had sepsis, and 146 (32.44%) patients had septic shock. Most (n = 358, 79.5%) patients had some degree of PS impairment (ECOG score ≥ 1). The mean SOFA score was 6.5 (± 3.26).

**Table 1:** Patient characteristics

Patient characteristics	All patients
Age (years)	59.60 ± 14.4
Gender (male sex)	248 (55.1)
ECOG-PS	
0	92 (20.44)
1	145 (32.22)
2	123 (27.33)
3	50 (11.11)
4	40 (8.89)
SAPS (points)	86.65 ± 16.69
SOFA on first day in ICU (points)	6.53 ± 3.26
Type of cancer	
Hematological malignancies	84 (18.67)
Locorregional solid tumor	112 (24.89)
Metastatic solid tumor	254 (56.44)
Solid tumor source	
Gastrointestinal	132 (29.33)
Genitourinary	40 (8.89)
Head and neck	39 (8.67)
Gynecological	61 (13.56)
Lung	26 (5.78)
Classification according to Sepsis-3	
Infection	16 (3.56)
Sepsis	288 (64)
Septic shock	146 (32.44)
Infection site	
Respiratory	96 (21.33)
Abdominal	89 (19.78)
Urinary	37 (8.22)
Organ support	
Mechanical ventilation	79 (17.56)
Renal replacement therapy	32 (7.11)
Vasopressor	343 (76.22)
Outcome data	
ICU length of stay (days)	5.7 ± 8.6
End of life decisions	99 (22)
ICU mortality	175 (38.9)
Hospital mortality	265 (58.9)

ECOG - Eastern Cooperative Oncology Group; PS - performance status; SAPS - Simplified Acute Physiology Score; SOFA - Sequential Organ Failure Assessment; ICU - intensive care unit. The results are expressed as the mean ± standard deviation or n (%).

**Figure 2:**
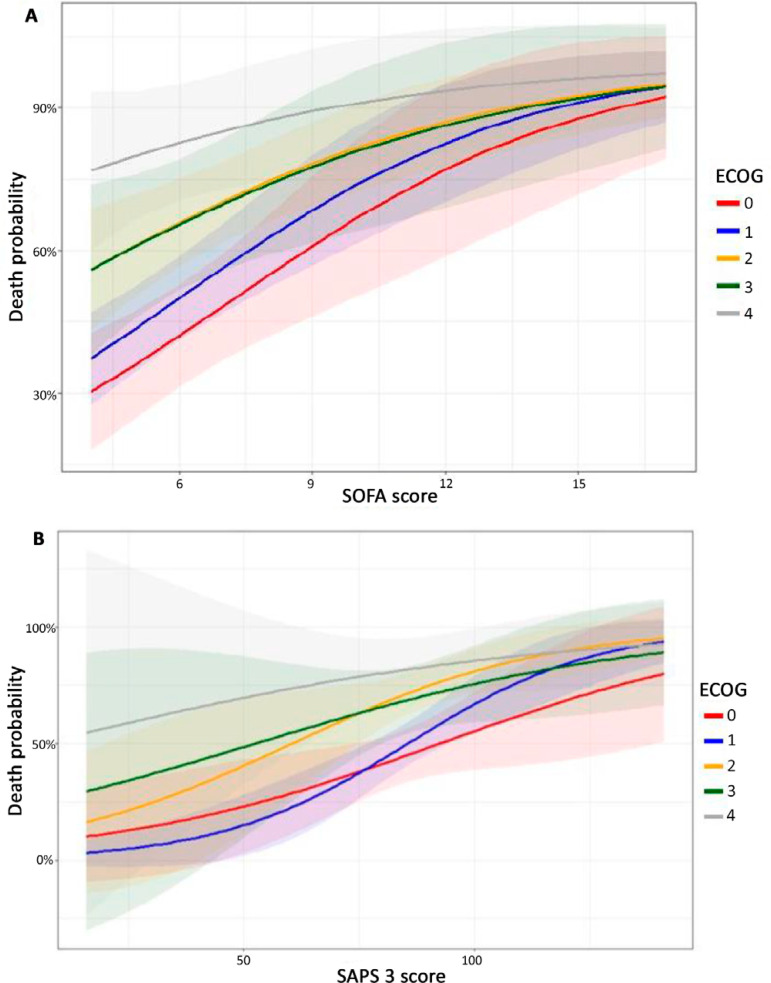
Hospital mortality among patients defined by the Eastern Cooperative Oncology Group performance status category according to the Sequential Organ Failure Assessment (A) and Simplified Acute Physiologic Score 3 (B). SOFA - Sequential Organ Failure Assessment; ECOG - Eastern Cooperative Oncology Group; SAPS 3 - Simplified Acute Physiologic Score 3.

In general, worse ECOG was associated with higher in-hospital mortality rates (Figure 2A). However, the PS influenced hospital mortality differently in patients with increasingly severe acute organ dysfunction. In patients admitted to the ICU with lower SOFA, PS clearly influenced in-hospital mortality. On the other hand, for those patients with higher SOFA, the PS did not seem to be a relevant predictor of hospital mortality (Figure 2A). There was no clear association between the PS and hospital mortality according to SAPS 3 (Figure 2B).

**Figure 3:**
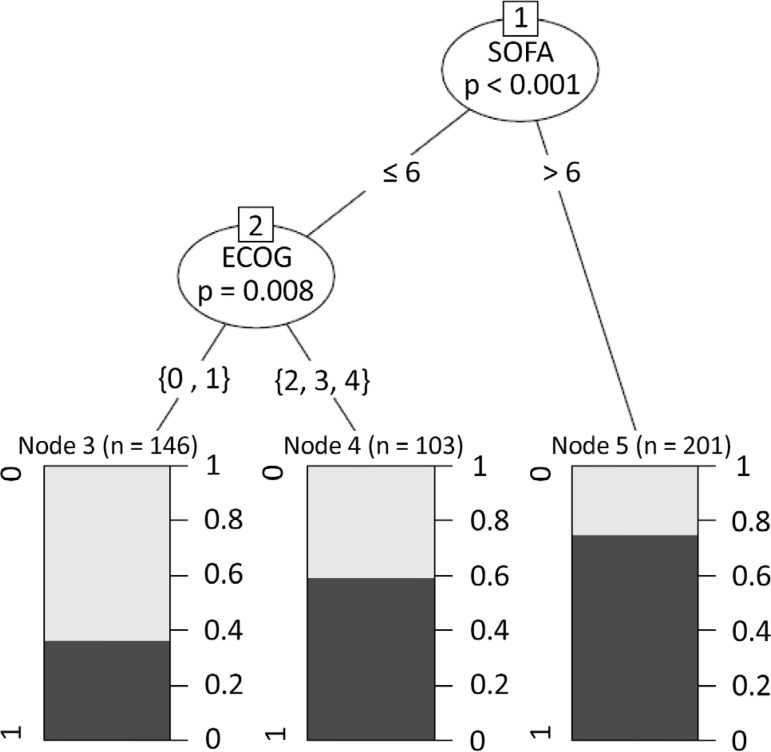
Relationships of Eastern Cooperative Oncology Group and Sequential Organ Failure Assessment scores to hospital mortality. SOFA - Sequential Organ Failure Assessment; ECOG - Eastern Cooperative Oncology Group.

The conditional inference analysis showed that the PS did not influence hospital mortality in all septic patients with cancer admitted to the ICU, and acute organ dysfunction was more relevant than the PS to the mortality among patients with SOFA > 6 (in-hospital mortality rate, 75%). In this group, there was no difference between patients with no or minor impairment (in-hospital mortality rate, 66%, n = 42/63) and those with moderate or severe impairment (in-hospital mortality rate, 79%, n = 110/139) (p *=* 0.06). Among patients with SOFA ≤ 6, the hospital mortality rate was higher among patients with moderate and severe PS impairment (ECOG two to four; 53%, n = 29/90) than among those with no or minor impairment (ECOG zero to one; 32%, n = 83/157; p = 0.002) (Figure 3).

We evaluated the interaction between PS impairment and SOFA score by the inclusion of their product (ECOG x SOFA) in the logistic regression, but there was no association with hospital mortality (p = 0.6).

There was no correlation between PS impairment and the SOFA score (rho: 0.01; p = 0.1)

## DISCUSSION

Our results suggest that the PS and acute organ dysfunction interact to affect hospital mortality in critically ill patients with cancer admitted to the ICU with suspected infection. For patients with lower SOFA, a worse PS impairment was associated with greater hospital mortality. However, for patients with higher SOFA, the PS did not seem to have a significant impact on hospital mortality.

In the present study, the sepsis in-hospital mortality rate was 59%, similar to rates found in other studies.^(1,4,5,8,9)^ Thus, the majority of critically ill patients with cancer and sepsis will die, regardless of ICU care. An understanding of the main factors associated with such high mortality is of paramount importance.

Previous researchers have described the PS as a prognostic factor for patients with cancer,^(4)^ specifically for those admitted with sepsis.^(5,10)^ Additionally, the ECOG PS scale is a fundamental tool used in clinical practice to guide treatment decisions for patients with cancer. The American Society of Clinical Oncology (ASCO) guidelines recommend against the use of chemotherapy, major surgery, and radiation therapy in patients with ECOG > 2.^(11)^ Therefore, the evaluation of the PS is of paramount importance in critically ill patients with cancer to define their management (i.e., full code status, limited ICU treatment trial, or palliative care measures).^(12,13)^

As many studies have shown that poor initial physiological scores and poor functional status are associated with mortality in critically ill patients with cancer,^(4)^ we hypothesized that PS would influence hospital mortality in septic cancer patients according to severity of the patient at admission. In a retrospective analysis, Zampieri et al.^(14)^ found that the combined consideration of the PS and the SAPS 3 enhanced discriminative ability in predicting hospital mortality. In this study, we evaluated the influences of the PS and the SOFA score at admission on hospital mortality in septic patients. We chose the SOFA score instead of the SAPS 3 because intensivists tend to make decisions based on the severity of acute organ failure, and the sepsis definition is based on this score.^(7,15)^ Thus, we suggest that acute organ dysfunction does not seem to be a mediator of the effect of the PS impairment on outcomes. A PS impairment was associated with worse outcomes only in the less severely ill patients (i.e., those with SOFA ? 6), whereas acute organ dysfunctions were independently associated with higher mortality rates in more severely ill patients (i.e., those with SOFA > 6).

This study has some limitations. First, as a single-center retrospective analysis, it was subject to local bias. Second, the small sample size prevented us from drawing solid conclusions. Third, as the ECOG classification was based on previous PS reported by family members at the time of ICU admission, the degree of patient impairment could have been overestimated. Fourth, end-of-life decisions were made during the ICU stays of 22% of patients in the cohort, which probably influenced hospital mortality. Fifth, it was not possible to adjust our model for the Charlson comorbidity index because we did not have this variable in our database.

## CONCLUSION

Our study findings suggest that the performance status impairment influences hospital mortality in critically ill patients with cancer differently according to the severity of acute organ dysfunction. For patients with high Sequential Organ Failure Assessment at the time of admission, the performance status had no influence on hospital mortality. For those with less severe acute organ dysfunction on admission, moderate and severe performance status impairment negatively influenced hospital mortality. Further studies should be conducted to assess the influence of the performance status in different strata defined by acute organ dysfunction to aid in the decision making for critically ill patients with cancer.
